# Diffusion-weighted imaging of the breast—a consensus and mission statement from the EUSOBI International Breast Diffusion-Weighted Imaging working group

**DOI:** 10.1007/s00330-019-06510-3

**Published:** 2019-11-30

**Authors:** Pascal Baltzer, Ritse M. Mann, Mami Iima, Eric E. Sigmund, Paola Clauser, Fiona J. Gilbert, Laura Martincich, Savannah C. Partridge, Andrew Patterson, Katja Pinker, Fabienne Thibault, Julia Camps-Herrero, Denis Le Bihan, On behalf of the EUSOBI international Breast Diffusion-Weighted Imaging working group

**Affiliations:** 1 Department of Biomedical Imaging and Image-guided Therapy, Division of Molecular and Gender Imaging, Medical University of Vienna/Vienna General Hospital, Wien, Austria; 2 Department of Radiology, Radboud University Medical Centre, Nijmegen, Netherlands; 3 Department of Radiology, The Netherlands Cancer Institute, Amsterdam, Netherlands; 4 Department of Diagnostic Imaging and Nuclear Medicine, Kyoto University Graduate School of Medicine, Kyoto, Japan; 5 Department of Radiology, New York University School of Medicine, NYU Langone Health, Ney York, NY 10016 USA; 6 Department of Radiology, University of Cambridge, Cambridge Biomedical Campus, Cambridge, United Kingdom; 7 SOC Radiodiagnostica, ASL AT, Asti, Italy; 8 Department of Radiology, University of Washington School of Medicine, Seattle, Washington USA; 9 MSKCC, New York, NY 10065 USA; 10 Institut Curie, Paris, France; 11 Ribera Salud Hospitals, Valencia, Spain; 12 NeuroSpin, Frédéric Joliot Institute, Gif Sur Yvette, France

**Keywords:** Diffusion magnetic resonance imaging, Biomarkers, Breast, Breast neoplasms, Consensus

## Abstract

The European Society of Breast Radiology (EUSOBI) established an International Breast DWI working group. The working group consists of clinical breast MRI experts, MRI physicists, and representatives from large vendors of MRI equipment, invited based upon proven expertise in breast MRI and/or in particular breast DWI, representing 25 sites from 16 countries. The aims of the working group are (a) to promote the use of breast DWI into clinical practice by issuing consensus statements and initiate collaborative research where appropriate; (b) to define necessary standards and provide practical guidance for clinical application of breast DWI; (c) to develop a standardized and translatable multisite multivendor quality assurance protocol, especially for multisite research studies; (d) to find consensus on optimal methods for image processing/analysis, visualization, and interpretation; and (e) to work collaboratively with system vendors to improve breast DWI sequences. First consensus recommendations, presented in this paper, include acquisition parameters for standard breast DWI sequences including specifications of *b* values, fat saturation, spatial resolution, and repetition and echo times. To describe lesions in an objective way, levels of diffusion restriction/hindrance in the breast have been defined based on the published literature on breast DWI. The use of a small ROI placed on the darkest part of the lesion on the ADC map, avoiding necrotic, noisy or non-enhancing lesion voxels is currently recommended. The working group emphasizes the need for standardization and quality assurance before ADC thresholds are applied. The working group encourages further research in advanced diffusion techniques and tailored DWI strategies for specific indications.

Key Points

*• The working group considers breast DWI an essential part of a multiparametric breast MRI protocol and encourages its use.*

*• Basic requirements for routine clinical application of breast DWI are provided, including recommendations on b values, fat saturation, spatial resolution, and other sequence parameters.*

*• Diffusion levels in breast lesions are defined based on meta-analysis data and methods to obtain a reliable ADC value are detailed.*

**Electronic supplementary material:**

The online version of this article (10.1007/s00330-019-06510-3) contains supplementary material, which is available to authorized users.

## Introduction

Diffusion-weighted imaging (DWI) is emerging as a key imaging technique to complement dynamic contrast-enhanced magnetic resonance imaging (DCE-MRI) of the breast. DWI can be used to distinguish between benign and malignant breast lesions [1–13], stratify in situ from invasive disease [14–18], and potentially predict the response to and monitor the effect of neoadjuvant treatment over time [19–25]. Excellent reviews can be found in the literature; however, there is still no clear consensus within the literature on where and how breast DWI should be applied.

A major strength of DWI is that the apparent diffusion coefficient (ADC) can be derived from it, providing a quantitative measure of observed diffusion restriction. We use the term “restriction” because it is widely used in the breast DWI literature. However, the term refers specifically to water diffusion in enclosed spaces, whereas ADC is sensitive to multiple processes occurring in human tissue (genuine restricted intracellular motion, compartmental exchange due to cell membrane permeability, hindered extracellular motion, water bounding to macromolecules, blood microcirculation, etc.) [26]. Thus, the term “hindrance” is also used in literature to describe diffusion effects and might be a more accurate description. Advanced protocols and analyses (as discussed later in this statement) may be used to (partly) disambiguate these effects. However, the focus of this statement is on increasing the translation and standardization of DWI for breast cancer evaluation using the established ADC quantification.

A current challenge is the large variability in results, i.e., specificity, sensitivity and thresholds, reported for ADC in distinguishing between benign and malignant breast lesions [9]. A less evident challenge is the inconsistent image quality due to different MRI system capabilities as well as equipment and imaging sequences/protocols contributing to a perceived limited usefulness of DWI in clinical practice. These challenges, coupled with the lack of prospectively validated thresholds for supporting diagnostic decisions, have prevented DWI from becoming an established measure that can be easily incorporated into the Breast Imaging Reporting and Data System (BI-RADS, [27]). Nevertheless, DWI has been incorporated (albeit in a qualitative way) into the Prostate Imaging Reporting and Data System (PI-RADS) [28].

In spite of the diversity in DWI protocols (e.g., the lack of standardization and the presence of artifacts), ADC estimation and interpretation methods across clinical sites [9, 29], and composition of breast lesions in the studies [21, 30–33], there is common agreement that DWI is sensitive to tissue microstructure and cellularity and provides quantitative information that can be used for lesion characterization. The improved lesion characterization can reduce the number of unnecessary biopsy recommendations [6, 7, 34, 35], which has been further validated in a multicenter trial [13]. The quantitative nature of ADC measures combined with relatively short acquisition times, typically in the order of 2–4 min but not exceeding 5 min, makes it an ideal imaging biomarker candidate [36]. Consequently, there is great interest to improve the generalizability and reproducibility of breast DWI across institutions and imaging platforms. Moreover, although technically challenging, DWI protocol standardization between different systems and vendors has been achieved in the breast and other organs [21, 24, 37–39].

### The International Breast DWI Working Group

To address the above-described issues and support standardized implementation of DWI, promote its clinical use, and facilitate its adoption as an integral part of breast MRI interpretation within BI-RADS, an International Breast DWI Working Group was established by the European Society of Breast Radiology (EUSOBI). Members of the working group were invited based upon proven expertise in breast MRI and/or breast DWI, representing 25 sites from 16 countries. Within the working group, a scientific committee was appointed. The full composition of the working group is given in Appendix 1.

The primary goals of the International Breast DWI working group are:

- To promote the integration of DWI into clinical practice by issuing consensus statements and initiate collaborative research where appropriate
- To define standards and provide practical guidance for clinical application of DWI
- To develop a standardized and translatable multisite multivendor quality assurance protocol, especially for multisite research studies
- To find consensus on optimal methods for image processing/analysis, visualization, and interpretation
- To work collaboratively with system vendors to improve breast DWI sequences

Members of the working group were first invited to provide information on their protocols with details of acquisition and processing parameters. This revealed substantial heterogeneity of applied DWI protocols, even among experts. The members of the Scientific Committee were then surveyed with a list of points on basic requirements, acquisition parameters, hardware, quality control, analysis and reporting to define the minimum standards for breast DWI, along with technical recommendations to meet these standards.

In this statement, we report the requirements for breast DWI that obtained general consensual agreement. The items that reached more than 80% consensus are shown in the Supplementary Material. This general guidance should be used to provide consistency across clinical trials and in clinical practice.

The group acknowledges that there are many items that may influence both the acquisition and reporting of breast DWI that are not discussed in detail in this statement. Those items did not reach consensus at this stage, requiring further discussion and/or more extensive research. Some of these issues include field strength effects, diffusion gradient waveform dependence, image registration, eddy current distortion and compensation, and gradient nonlinearity correction. Each of these topics has received attention in the breast DWI literature (see below), providing a roadmap for their incorporation in a consensus process. In that spirit, further work is intended from subgroups of our working group to methodically address such items, especially regarding clinical implementation, standardization and quality control, and advanced diffusion MRI methods. The results of these efforts are to be presented in a second round of topical guidelines.

## When to perform breast DWI?

Breast DWI may be part of any multiparametric breast MRI protocol independent of the clinical indication for MRI, as DWI improves the characterization of lesions detected by contrast-enhanced MRI irrespective of the indication [6, 7, 34, 35, 40]. This includes breast MRI performed for pre-operative staging of known breast cancer (ipsi- and contralateral), monitoring neoadjuvant systemic therapy, evaluating carcinomas of unknown primary origin, resolving equivocal findings from other imaging modalities, and solving problem. For all these indications, breast DWI is considered an important addition to DCE-MRI to improve specificity, with the aim of reducing the number of recalls and biopsies of benign breast lesions. A more detailed description of current indications for breast MRI is found in existing guidelines from the EUSOBI, European Society of Breast Cancer Specialists, and American College of Radiology [41–43].

The working group highlights a special case: the application of breast DWI for breast cancer screening. There is not yet enough evidence to recommend for or against the inclusion of DWI in screening protocols. While DWI may improve lesion classification in this setting, its use should be balanced against the limited frequency of abnormal findings (because in screening, most exams are normal). For example, rather than performing DWI in all screening examinations, in order to prevent unnecessary biopsies, a multiparametric MRI including DWI could be performed as a secondary evaluation in women with ambiguous findings at the screening test.

### Could breast DWI be used as a stand-alone test for breast cancer detection?

In general, even though breast DWI has high specificity for lesion characterization, the sensitivity of DCE-MRI still exceeds that of breast DWI [41–45], albeit the sensitivity of DWI alone may be equal to or even higher than that of commonly used screening techniques such as mammography and ultrasound. Thus, currently, its use for cancer detection as part of an unenhanced MRI examination requires further investigation and should be considered only in cases where DCE-MRI is not accessible or not appropriate. In particular, DWI may be a valuable alternative option in patients with contraindications to gadolinium-based contrast agents (such as patients with severe kidney dysfunction at risk of nephrogenic systemic fibrosis and patients with a previous acute reaction to a gadolinium-based contrast agent [44]).

### Is breast DWI helpful in the assessment of implants?

Breast DWI is not helpful in the assessment of breast implant integrity, which relies on other dedicated non-contrast techniques such as T2-weighted imaging and silicone specific sequences and thus is not needed for this purpose. It is, however, as useful as in any other indication for lesion classification in patients with implants.

## What are the minimal technical requirements for breast DWI?

The working group found consensus on a minimal set of acquisition parameters to be met in clinical practice. Adherence to these minimal requirements should improve the comparison of ADC values from site to site, which is an important step towards the generalizability required to eventually incorporate ADC quantification into standardized guidelines (e.g., BI-RADS).

### Which hardware should be used for breast DWI?

Breast DWI should be undertaken in a closed bore magnet with field a strength of 1.5 T or more. The gradient hardware should be capable of reaching a maximum gradient strength of at least 30 mT/m, and the use of a dedicated breast coil with at least four channels is strongly recommended. When possible, DWI should be performed before DCE-MRI, as the presence of contrast agents may physically reduce measured ADC values. It should be mentioned, however, that no significant effect has been shown on overall diagnostic performance when DWI is obtained after contrast administration as long as fat suppression using the STIR technique is avoided [5, 9, 45].

### What sequence type and parameters are recommended for breast DWI?

The recommended minimal requirements for breast DWI are shown in Table 1. The working group acknowledges that for different MRI systems, different parameters might need to be adapted in order to obtain the best possible results. Therefore, Table 1 should be regarded as a guide rather than as a checklist for optimizing a DWI sequence. For instance, the axial orientation was motivated by anatomy and ease of interpretation more than technical optimization, which depends on the coil configuration. Specifying more parameters is beyond the scope of this paper but will be addressed at a later stage.

**Table 1:** Minimum requirements for diffusion-weighted-imaging of the breast as per working group consensus

Acquisition parameter	Minimum requirement	Specific remarks
Type of sequence	EPI based	
Orientation	Axial	
Field of view	The field of view should cover both breasts	Covering the axillary region is not mandatory
In-plane resolution	≤ 2 × 2 mm^2^	Acquired physical resolution, before reconstruction, and reconstructed resolution
Slice thickness	≤ 4 mm	
Number of *b* values	2	More is optional
Lowest *b* value	0 s/mm^2^	In practice as close to 0 as possible, but not exceeding 50 s/mm^2^
High *b* value	800 s/mm^2^	
Fat saturation	Required	SPAIR is recommended
TE	Minimum possible by the system and choice of parameters	Optimize the rBW to obtain minimum TE. The reduction in SNR by increasing the rBW is usually compensated for by the shortening of TE
TR	≥ 3000 ms	
Acceleration	Parallel imaging (factor ≥ 2)	Reduces distortion, loss in SNR can be counterbalanced by increasing the number of excitations
Post-processing	Generation of ADC maps is required	Standard ADC is calculated as ADC = ln (*S*_low_/*S*_high_) / (*b*_high_−*b*_low_) where *S*_low,high_ are the image signal values obtained with *b* values *b*_low,high_

It should be understood that the suggested in-plane resolution also corresponds to the physical resolution of the native images. Acquisition parameters aimed at reducing acquisition times or improving image quality (e.g., phase resolution) may introduce significant differences between the prescribed image resolution and the actual acquired resolution. Reconstruction and post-processing (e.g., interpolation) may also alter the native image resolution. Support from vendor technicians may be helpful when in doubt

*EPI*, echo-planar imaging; *rBW*, receiver bandwith; *SPAIR*, spectrally adiabatic inversion recovery; *TE*, echo time; *TR*, repetition time

Single-shot or multi-shot echo-planar imaging (EPI) is regarded as baseline techniques for DWI acquisition. EPI has a wide breadth of applications and a correspondingly deep library of tools to address its shortcomings in image quality. Examples are nonlinearities in diffusion gradients, eddy current or magnetic field–induced image distortions, and motion. Gradient nonlinearities have been identified as a major source of inaccuracy for breast DWI [46], and prescriptions for correction have been offered [47]. Eddy current and inhomogeneous field distortions are well-known issues for diffusion MRI with evolving solutions. Inhomogeneous field distortion correction [48] and motion correction strategies [49] have been applied to breast DWI. Multi-shot EPI, especially within the RESOLVE sequence framework [50] has the potential to reduce susceptibility-induced geometric distortion and motion artifacts, at the expense of the acquisition time. However, corrective elements for breast DWI acquisitions are currently not sufficiently standardized to recommend a specific approach and will be addressed in future work of our working group. Nonetheless, the group made a recommendation on using an acceleration factor of 2, which reduces eddy current effects (and distortion). Vendor-based standard product pulse sequences with inline workflow elements (eddy current correction, gradient nonlinearity correction) may be used, but the optimal correction strategies are yet unknown.

### Which *b* values should be chosen for breast DWI?

**Fig. 1:**
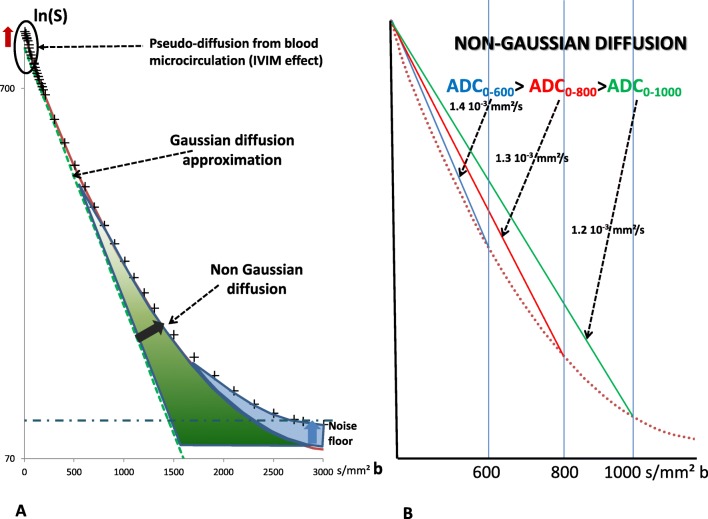
Diffusion MRI signal decay versus *b* value. **a** The diffusion signal attenuation (logarithmic signal attenuation versus *b* value) follows a straight line when diffusion is free (dotted green line). In tissues, hindrance/restriction of water diffusion by many microscopic obstacles results in a reduced rate of raw signal attenuation and a curvature (cross symbols) which increases with the *b* value. The ADC value (for instance, calculated from *b* = 0 and 800 s/mm^2^) is, thus, lower than the free diffusion coefficient, due to these combined effects. At high *b* values, the signal may further reach a “noise floor” and no more diffusion information can be extracted. Conversely, at very low *b* values, the signal attenuation rate can be elevated, because blood circulation in the random capillary network mimics diffusion (pseudo-diffusion, which is referred to as intravoxel incoherent motion (IVIM)). **b** The conceptual diagram illustrates the need for standardization of the applied *b* values. As the ADC value is calculated assuming a linear signal decay while the actual signal attenuation is curved, the ADC value decreases when using higher *b* values, as more restriction/hindrance effects are integrated into the ADC value; this illustrates the importance of using common *b* values for standardization

The choice of *b* values is critical. Due to the non-Gaussian nature of water diffusion in tissues (which results in a curvature of the DWI signal attenuation plot across *b* values), the ADC value is highly dependent on the choice of *b* values, with the ADC values getting smaller as larger *b* values are used (Fig. 1). Higher *b* values may increase the specificity of DWI [11] and also lead to a decreased signal-to-noise ratio. A high *b* value of 800 s/mm^2^ was chosen by the group as a good compromise for standardization. This value can be established theoretically [51] and is backed up by empirical evidence [9].

### In how many directions should breast DWI be acquired?

The number of directions to acquire diffusion measurements from which the final ADC is calculated can vary from one direction to more than 6 directions for diffusion tensor imaging (DTI). Using several different diffusion directions allows calculation of an average ADC and reduces the influence of anisotropy in the tissue, thus improving generalizability. The “3 orthogonal directions” method (commonly available clinically and less time consuming than the 6 directions scheme required for DTI) provides an approximate isotropic weighting (a.k.a. pseudo trace-weighting contrast as an approximation to the true trace which can be obtained from the eigenvalue average derived from the full diffusion tensor). This 3 directions approach may also mitigate residual variations of *b* values across directions. However, variable eddy current interactions with the EPI readout train may result in different image distortions according to the diffusion-encoding direction, which, when averaged over the 3 directions, can confound image quality and reduce sensitivity to small lesions. Alternatively, acquisitions along only 1 direction but using gradient pulses simultaneously on 2 or 3 axes (sometimes called “diagonal”) allow high *b* values to be reached using shorter TEs, thus increasing SNR and reducing acquisition time compared with a sequential 3 direction acquisition. Diagonal diffusion gradients, however, would introduce variability in the presence of diffusion anisotropy known to exist in fibroglandular breast tissue. Given these mixed advantages, the compromise recommended is to utilize 3 orthogonal directions. This scheme is universally available and provides approximately isotropic contrast. However, we must emphasize that this approach can introduce non-negligible error and bias in the estimation of diffusion kurtosis imaging-derived indices of the breast and is thus not appropriate for this application [52].

### How can high image quality and SNR be ensured?

**Fig. 2:**
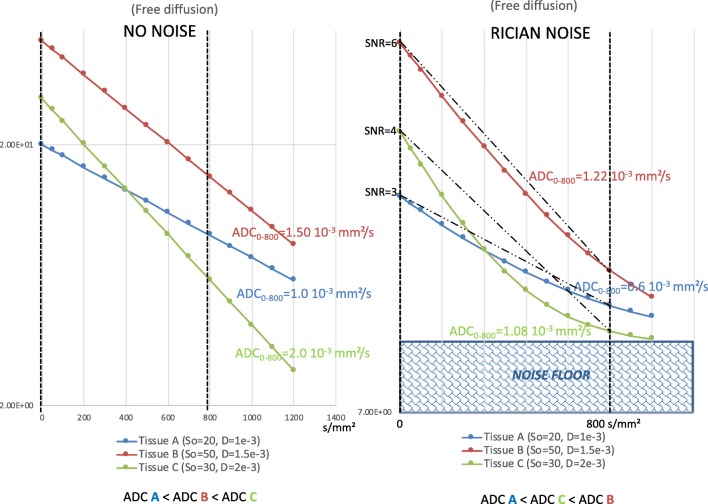
An example of the effect of the noise floor on the observed ADC values. For three hypothetical tissues with free diffusion (no hindrance/restriction effect for simplicity), in the absence of noise (left), the slope gives the ADC values of 1.0, 1.5, and 2.0 10^-3^ mm^2^/s, respectively, for tissues A, B, and C. However, in the presence of noise (right), the noise floor leads to curvature of the signal decay. As a result, the ADC values change. In this example, we measured 0.6, 1.22, and 1.08 10^-3^ mm^2^/s, respectively, for tissues A, B, and C. It should be noted that the observed ADC value in tissue B is now higher than that in tissue C. This order no longer reflects diffusion, but the amount of signal at *b* = 0 s/mm^2^, which depends solely on tissue magnetization properties (A has the lowest signal level and B the highest). Consequently, a low SNR may result in misclassification of the diffusion level of a lesion. S0 indicates the signal intensity at *b* = 0 s/mm^2^, and *D* = diffusion coefficient

The signal-to-noise ratio (SNR) of DWI is also important, especially when a high *b* value is used as ADC values may be largely underestimated (mimicking malignant tissues) when the SNR is low (Fig. 2). This requires that a sufficiently high SNR is achieved. One qualitative requirement in that vein is that fibroglandular tissue (FGT) (if present) should be well depicted on low *b* value images. Quantitatively, while the term “sufficient SNR” has yet to be defined in DWI of the breast, acquisition with multiple excitations for averaging, especially at *b* = 800 s/mm^2^, will be necessary to reach this goal. Unfortunately, the working group acknowledges that there are currently no universal methods to quantify SNR easily in a clinical setting, especially with the use of multichannel coils. In that setting, prescriptions exist for accurate SNR determination using difference methods [53] for average noise assessment or the Kellman approach [54] for spatially varying SNR, but they require additional acquisitions and advanced reconstruction that are not sufficiently ubiquitous to reach a consensus recommendation. Consequently, before implementing a new DWI sequence in routine clinical care, it is advisable to first test the sequence and assess image quality qualitatively. Ideally, quality control should be performed using dedicated calibrated phantoms with known ADC values, especially for multicenter studies (see below). Such phantoms will also allow the correct application of diffusion gradients (i.e., *b* values across axes) to be checked. In clinical practice, it remains important to check regularly whether the obtained diffusion images have a sufficient SNR and are reasonably free of structural artefacts that might hamper interpretation. While previous studies have addressed quality controls in DWI in general [55–57], one study investigated in detail factors influencing SNR and quantitative ADC measures in breast DWI [58]. The results of this study corroborate the hypothesis that scanners should be adequately characterized in breast DWI. Quality control is also required after the maintenance of the MRI system and after each upgrade of hard- and/or software.

Apart from low SNR, a commonly encountered problem is insufficient fat suppression, which leads to ghosting and potentially to underestimation of ADC values. Spectral attenuated inversion recovery (SPAIR), which employs both T1 contrast and spectral selectivity, is recommended given a moderate preference in literature in comparison with short tau inversion recovery (STIR) [59, 60] and based on the consensus among the panel. However, if unsuccessful, STIR may be an acceptable alternative as this technique is independent from *B*_0_ field inhomogeneity [61].

While not listed in Table 1, diffusion time is a parameter that may significantly impact ADC values. Although it is generally hidden to users (gradient pulse time course is usually not reported), with conventional sequences, diffusion time is approximately equal to TE/2. The degree of diffusion restriction (or hindrance) decreases at short diffusion times (increasing the ADC) [62] and it is important to be aware of this parameter as some vendors are now proposing stronger gradient hardware that allows shorter diffusion times. However, on most clinical MRI scanners, achievable diffusion times cannot be shortened below ~ 25 ms, which leads to comparable results between machines.

## How to evaluate and interpret breast DWI?

### How are lesions identified on breast DWI?

In a multiparametric breast MRI protocol, lesion detection should be primarily based on evaluation of the contrast-enhanced sequences, which can be aided by inspection of the *b* = 800 s/mm^2^ images. On the *b* = 800 s/mm^2^ images, cancers are typically hyperintense given adequate baseline T2 signal. As the basic DWI sequence is T2-weighted, lesions with higher water content (e.g., cysts, myxoid fibroadenoma, highly proliferative cancer) will show a high signal on low *b* value images and may retain a (relatively) high signal on high *b* value images. Consequently, a high signal on *b* = 800 s/mm^2^ images may be due to a very high T2 signal (commonly referred to as T2 shine-through, although physically misleading) or true diffusion restriction with little signal decrease of a moderately high T2 signal. Cross-correlation of *b* = 800 s/mm^2^ image findings with quantitative ADC maps usually allows discrimination between these instances. Therefore, DWI analysis requires the evaluation of raw DWI data and ADC maps together.

**Fig. 3:**
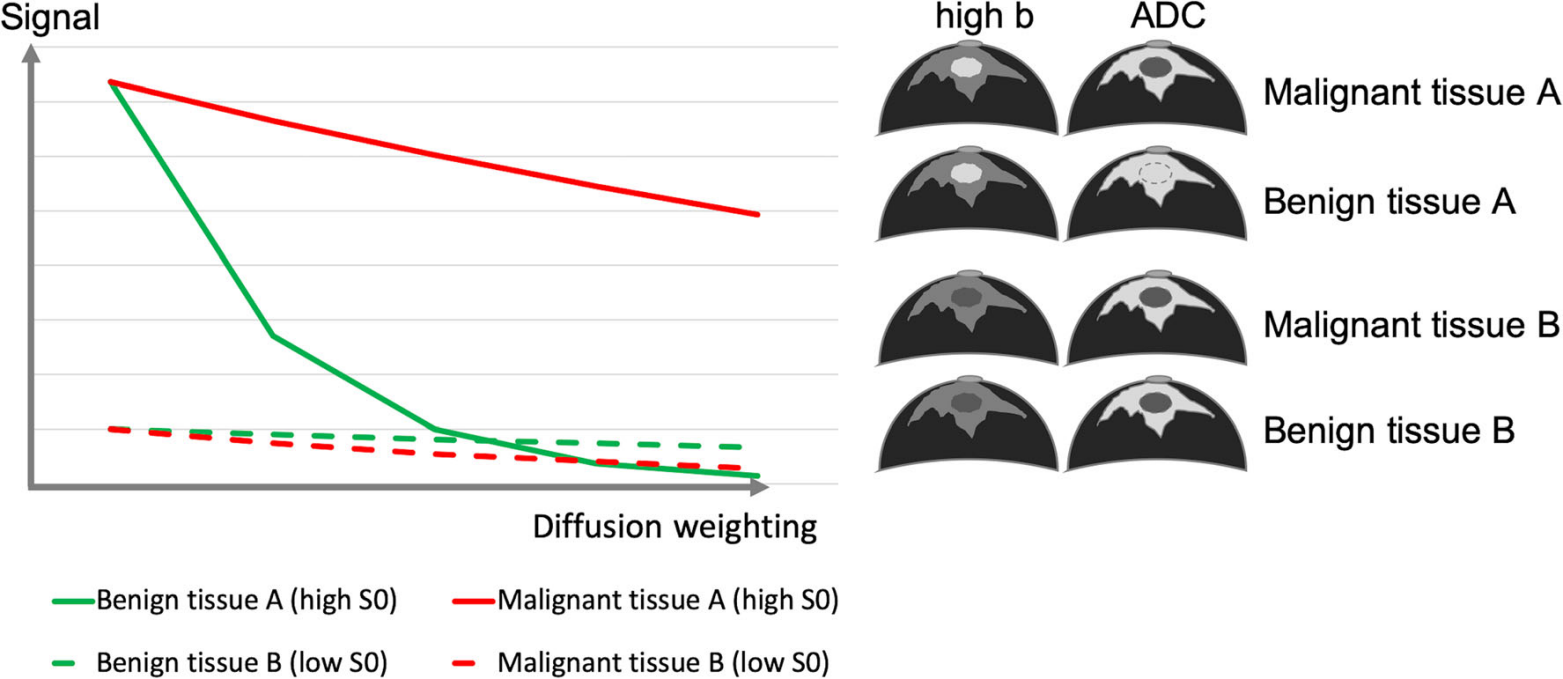
Observed signal decay in benign and malignant lesions depending on baseline T2 signal. Tissues and lesions with a very low water content (e.g., fibrotic parenchyma, scars, low cellular cancers with extensive desmoplastic stromal fibrosis) may not be visible on high *b* value images and present with an artefactual low signal decay leading to low ADC values. S0 indicates signal intensity at *b* = 0 s/mm^2^

On the other hand, tissues and lesions with a very low water content (e.g., fibrotic parenchyma, scars, some invasive lobular cancers) may demonstrate a very low signal at *b* = 0 s/mm^2^ (and thus, also on *b* = 800 s/mm^2^). Thus, these lesions will be difficult to visualize. In these lesions, the measurement of diffusion-dependent signal loss may not be possible, thus preventing an accurate assessment of diffusion level (Fig. 3). ADC in this condition (that can be referred to as signal blackout) will be low but will not reflect a true diffusion restriction (Fig. 3).

### Can lesion location, size, and morphology be assessed on DWI images?

Assessment of location, size, and morphology of lesions is possible on DW images, even though this is limited by a spatial resolution that is inferior to that found in anatomical and contrast-enhanced sequences, and when available, these should be interpreted together. Morphologic assessment on breast DWI may be reported when it is discrepant with other sequences. Lesions can be categorized as foci, masses, or non-mass lesions. For masses, shape (round, oval, irregular) and internal signal pattern (homogeneous, heterogeneous, rim) can be reported while in non-mass lesions, distribution (focal, regional, linear, segmental) and internal signal pattern (homogeneous, heterogeneous) can be reported.

### How should the ADC value be measured after DWI acquisition?

The pixel-wise parametric ADC maps enable quantitative assessment of diffusion restriction or hindrance, which should always be measured. The ADC value is obtained by drawing a region of interest (ROI) on the lesion on the ADC map (or the *b* = 800 s/mm^2^ image when the workstation allows propagation of the ROI to the ADC map). The ROI should fall completely within the lesion, contain at least 3 voxels and avoid both artifacts and necrotic or hemorrhagic parts of the lesion. This implies that lesions of 6 mm or larger in the axial plane are evaluable with DWI, albeit for small lesions, partial volume effects should be taken into account. While there was no consensus from our group on the size of the ROI to be used (whole lesion or focused ROI), literature suggests that selecting the lowest ADC value within the lesion (potentially reflecting the most active part of the lesion) might provide a more accurate discrimination between malignant and benign breast lesions [17, 29, 63, 64]. Due to the above described signal blackout, the ROI should fall within the enhancing part of the lesion (and hyperintense part on DW images) in order to avoid falsely low ADC values. Using a small ROI in the darkest part of the lesion on ADC maps is analogous to that used for analysis of DCE images. We thus suggest the use of a ROI placed on the darkest part of the ADC map, avoiding necrotic, noisy, or non-enhancing lesion voxels as the preferred method for measuring ADC values in order to reduce inter- and intra-reader variability and improve breast DWI consistency and comparability between sites. For such an ROI, the mean ADC value within the ROI should be reported, and it is suggested that the units should be in 10^-3^ mm^2^/s. Volumetric sampling of the whole lesion may be useful when the clinical indication is the evaluation of tumor response. In any case, the type of ROI (whole or focused) used for lesion assessment should be reported.

### How is the ADC value interpreted?

**Fig. 4:**
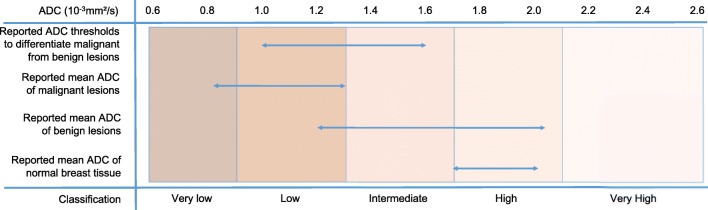
ADC thresholds and value ranges for malignant, benign, and normal tissue. In this graph, the lower horizontal arrows show the range of reported mean ADC values for normal breast tissue, benign, and malignant lesions. The top arrow shows the range of suggested thresholds to differentiate between benign and malignant lesions. Note that this graph simply lists ranges as taken from the original tables and no data pooling was performed. The color bars correspond to the diffusion levels that were defined and agreed upon by the working group in order to standardize the description of the diffusion values

Based on ADC measurements and lesion appearance, the proposed classification of diffusion level in lesions is very low, low, intermediate, high, and very high (Table 2 and Fig. 4). Figure 4 shows corresponding ADC ranges based on the most recent meta-analysis of studies evaluating DWI for the differentiation of benign and malignant lesions [10] and considering only those studies that were in line with our consensus *b* value suggestions. The ADC values provided in Table 2 are solely intended to describe lesions in an objective way according to their diffusion level. Lesion classification should not be based upon diffusion level alone but should always be performed in conjunction with all anatomical and functional information available from all other imaging data.

**Table 2:** Suggested diffusion level lexicon based on lesion appearance on *b* = 800 s/mm^2^ images, ADC maps, and ADC values

Diffusion level	Lesion appearance on *b*800 and ADC maps ^a^	ADC value range^b, c^
Very low	*b*800, very hyperintense ADC, very hypointense	≤ 0.9 × 10^-3^ mm^2^/s
Low	*b*800, hyperintense ADC, hypointense	0.9–1.3 × 10^-3^ mm^2^/s
Intermediate	*b*800, moderately hyperintense ADC, moderately hypointense	1.3–1.7 × 10^-3^ mm^2^/s
High (normal)	*b*800, no lesion visible ADC, no lesion visible	1.7–2.1 × 10^-3^ mm^2^/s
Very high	*b*800, hypo- or hyperintense ADC, hyperintense	> 2.1 × 10^-3^ mm^2^/s

*b*800 denotes *b* = 800 s/mm^2^ images

^a^The qualitative appearance is always relative to the background signal. Due to the very large variations in breast composition between women, the lesion appearance on *b*800 images may vary despite similar ADC values. Also, baseline or T2-related signal variations may cause variations of the lesion appearance on *b*800 images as given in the table, e.g., in case of T2 shine-through or signal blackout

^b^ADC values must be obtained under the following conditions: calculated from images acquired with the lowest *b* value as specified in Table 1 and 800 s/mm^2^ with fat suppression, before contrast injection, diffusion time > 25 ms, and good SNR in the lesion at *b* = 800 s/mm^2^

^c^The given values correspond to a generic population of patients. Adjustment may be needed to account for specific patient populations

**Fig. 5:**
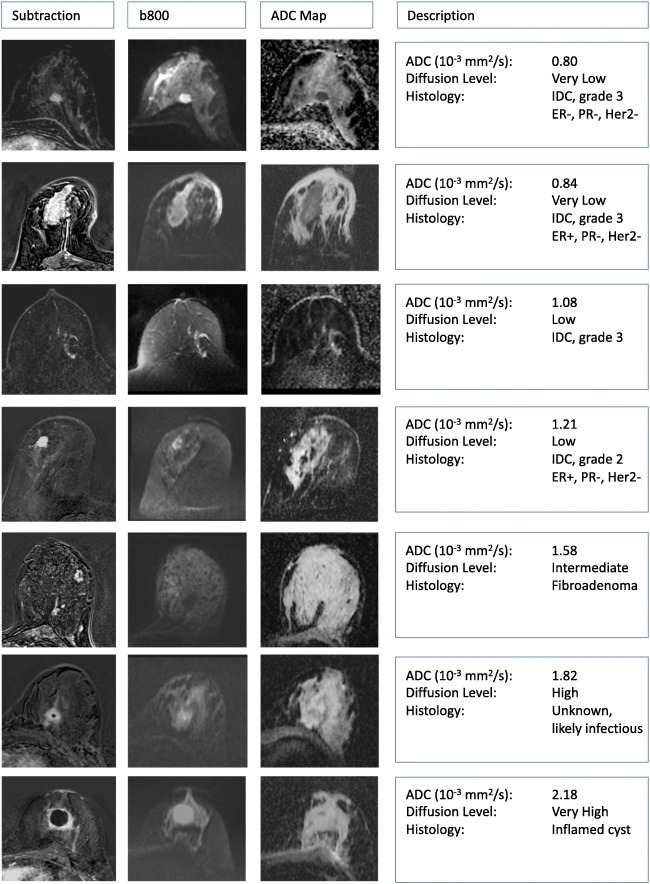
Clinical examples illustrating the diffusion levels presented in Fig. 4 and Table 2

Of the malignant lesions, invasive ductal and invasive lobular cancers, as well as DCIS with microinvasion, are usually associated with low to very low diffusion levels [16, 18, 65, 66]; pure ductal carcinoma in situ generally shows low or intermediate diffusion (16), whereas particularly invasive mucinous cancer may present with intermediate or even high diffusion levels [67]; and triple-negative cancers with extensive necrosis may also yield high or very high diffusion levels in the necrotic part [68]. Typical examples of benign and malignant lesions are presented in Fig. 5.

ADC measurements are prone to bias from adjacent noise regions (e.g., from voxels containing fat). These can be identified by unrealistically high (> 3 × 10-3 mm^2^/s), low (< 0.5 × 10-3 mm^2^/s) or even zero/negative ADC values. Unrealistic ADC values may require repositioning of the ROI.

### Why are specific quantitative cut-off values not provided?

Two goals of the working group are (a) to promote more widespread integration of DWI into clinical use with standardized acquisitions, increasing the global evidence base by inclusion of DWI in future clinical trials and (b) to derive quantitative discrimination levels supported by standardized and reproducible acquisitions to be incorporated into clinical diagnostic or prognostic guidelines. A practical reality is that these goals are symbiotic and achieving them is an unavoidably iterative process. Thus, the analysis and interpretation scheme based on both qualitative and preliminary quantitative features (ADC value ranges) presented here are preliminary. It is important to realize that these suggestions are not fully vetted by the procedures of quality control, reproducibility, and multisite concordance that underpin formalized quantitative imaging biomarkers (QIB) and thus may evolve with time. This process will also be amplified by the incorporation of experimental optimization (eddy current distortion correction, gradient nonlinearity correction) into the consensus process.

Consequently, the ADC values in Table 2 may be adjusted by our group in the future once more rigorous standardization guidelines are in place, based upon intended multicenter investigation. Similarly, the minimal consensus recommendations of this document may be further refined to minimize variability from protocol variation.

## Which steps are necessary for further implementation of quantitative breast DWI?

The recommendations outlined in this statement aim at improving inter-institutional protocol consistency, increasing homogeneity of reported data, and achieving a standardized assessment of the ADC values. The working group assigns high priority to establishing ADC as a QIB for broad diagnostic (lesion classification/aggressiveness) and prognostic (prediction of treatment response) use. Roadmaps for this process have been laid out by other consortia (e.g., Quantitative Imaging Biomarkers Alliance (QIBA, http://qibawiki.rsna.org/), Quantitative Imaging Network (QIN), and the International Society for Magnetic Resonance in Medicine Ad Hoc Committee on Standards for Quantitative MR). It is expected that the standardized acquisitions and evaluation suggested in this document will positively contribute to this process. Continuing efforts by (subgroups of) the working group to address the many other parameters that may influence acquisition and reporting of DWI will be guided by “metrology principles” of precision, repeatability, and reproducibility, tailored to breast imaging in particular.

As an example, physical phantoms are one key aspect of quality control [69]. The ideal phantom mimics the target tissue, morphologically and, in terms of MRI parameters, provides reproducible results and is either easily moved or reproduced at multiple sites. An existing system for oncologic imaging is based on the ice-water phantom [55, 70, 71], which provides established ADC values and built-in temperature control. Other phantoms specifically designed for breast MRI contain synthetic material (e.g., alkanes) mimicking the MR properties (T_1_, T_2_, ADC) of fibroglandular, adipose, and tumor tissue and built-in housings compatible with breast RF coils [72]. It has also been shown that high ADC phantoms such as solutions have utility in quantifying the difference between desired (nominal) and achieved (effective) *b* values [57]. In addition to ADC standardization, these systems have the added benefit of allowing quality control of the effectively applied *b* values and of fat suppression, key elements of breast DWI. While these systems involve a higher level of investment, they may be appropriate for the future stages of inter-site standardization envisioned by our working group.

Another element of quality control is test-retest repeatability. Within breast cancer patients, obtaining this information requires considerable commitment given the practicalities of multiple examinations, especially since normal values may be dependent on the menstrual cycle [31, 73, 74], and likely requires evaluation in research protocols. However, when available, it allows quantitative distinction of biologic changes from measurement imprecision, which is vital for the use of imaging biomarkers in clinical trials. Thus, research efforts are encouraged that collect repeatability data in patients wherever possible to build the evidence base.

## What is the clinical potential of advanced DWI techniques?

While the basic monoexponential diffusion model and resulting ADC values providing a simple and technically reproducible parameter are currently preferable in clinical practice, the working group explicitly acknowledges continuous developments in the field of DWI that hold promise and should be pursued in parallel with ADC standardization. For example, the heterogeneity of ADC values within lesions may be measured using histogram analysis and more advanced artificial intelligence techniques [75–77]. Furthermore, DWI techniques “beyond the ADC” [26], such as diffusion tensor imaging (DTI), which allows the analysis of diffusion anisotropy [78–83]; intravoxel incoherent motion (IVIM) imaging, which distinguishes between intravascular perfusion and extravascular microstructural diffusion components [84–89]; and non-Gaussian diffusion, which provides enhanced sensitivity to tissue complexity, for instance from the kurtosis model [11, 84–88, 90–93], may enhance the value of DWI. Hybrid combinations of these models have also been tested [11, 12]. However, such advanced methods will add constraints to the DWI acquisition protocol (multiple diffusion-encoding directions, multiple *b* values in the low and high range, etc.) resulting in longer acquisition times and the need for more sophisticated image processing tools. Currently, there is no reliable evidence regarding the clinical value superiority of advanced DWI techniques over standard ADC assessment [94]. Still, DWI users are explicitly encouraged to investigate more specific (or “tailored”) strategies aimed at screening (with/without the concurrent use of contrast-enhanced sequences), lesion characterization and staging, treatment monitoring, and prognosis.

## Conclusion

This statement details the first consensus on breast DWI created by the EUSOBI International Breast DWI working group. The working group considers breast DWI to be an essential part of a multiparametric breast MRI protocol. Basic requirements for routine clinical application of breast DWI are proposed, including recommendations on *b* values, fat saturation, spatial resolution, TR/TE, and considerations for ROI placement. The working group will focus our future efforts on expanding the technical recommendations of DWI protocols and the development of methods for quality control. Finally, the working group explicitly encourages research into more sophisticated advanced acquisition, modeling, and analysis approaches to further exploit the diagnostic and prognostic potential of diffusion-based breast imaging.

## Electronic supplementary material

ESM 1 (DOCX 34 kb)

## Abbreviations

EUSOBI: European Society of Breast Imaging
QIB: Quantitative imaging biomarker
SNR: Signal-to-noise ratio
SPAIR: Spectrally adiabatic inversion recovery
STIR: Short tau inversion recovery
