# Supplementary material for: Diffusion-weighted imaging of the breast—a consensus and mission statement from the EUSOBI International Breast Diffusion-Weighted Imaging working group
Source: Eur Radiol. 2019 Nov 30;30(3):1436–50. doi: 10.1007/s00330-019-06510-3 (PMC7033067; doi:10.1007/s00330-019-06510-3)
Supplement: Supplementary file 1 — (DOCX 34 kb) [file 330_2019_6510_MOESM1_ESM.docx]

**Appendix 1:** **EUSOBI International Breast Diffusion-Weighted Imaging Working Group**

Members of the working group consist of clinical breast MRI experts, MRI physicists and representatives from vendors of MRI equipment (Canon Medical Systems, GE Healthcare, Philips Healthcare, and Siemens Healthineers), representing 25 sites from 16 countries. Within the working group a scientific committee was appointed. The working group met in March 2017 during the European Congress of Radiology (ECR) in Vienna, in Berlin in September 2017 during the EUSOBI Annual Meeting, and in March 2018 during the ECR in Vienna. Additional less formal meetings also took place within the Scientific Committee.

**Chairs:** Julia Camps-Herrero and Denis Le Bihan

**Scientific Committee:**

- Pascal Baltzer
- Julia Camps-Herrero
- Paola Clauser
- Fiona Gilbert
- Mami Iima
- Denis Le Bihan
- Ritse Mann
- Laura Martincich
- Savannah Partridge
- Andrew Patterson
- Katja Pinker
- Eric Sigmund
- Fabienne Thibault

**Members:**

| **Firstname** | **Lastname** | **Place of Work** | **City** | **Country** |
| --- | --- | --- | --- | --- |
| Oshaani | Abeyakoon | Addenbrookes | Cambridge | United Kingdom |
| Akira | Adachi | Canon medical | Tochigi | Japan |
| Marina | Alvarez | Hospital Reina Sofia | Córdoba | Spain |
| Alexandra | Athanasiou | MITERA Hospital | Athens | Greece |
| Daly | Avendaño | San Jose Tec | Monterrey | Mexico |
| Sarah | Bacon | University of Leeds | Leeds | United Kingdom |
| Corinne | Balleyguier | Gustave Roussy | Villejuif | France |
| Pascal | Baltzer | Medical University of Vienna | Vienna | Austria |
| Tone F. | Bathen | NTNU | Trondheim | Norway |
| David | Buckley | University of Leeds | Leeds | United Kingdom |
| Michael | Burke | General Electric |  | United States of America |
| Julia | Camps Herrero | Hospital de la Ribera | Alzira | Spain |
| Luca A. | Carbonaro | IRCCS Policlinico San Donato | Milan | Italy |
| Paola | Clauser | Medical University of Vienna | Vienna | Austria |
| Maya | Cohen | Beillinson Hospital | Tel-Aviv | Israel |
| Cedric | de Bazelaire | Paris Diderot University | Paris | France |
| Matthias | Dietzel | University Hospital Erlangen | Erlangen | Germany |
| Monique | Dorrius | University Medical Center Groningen | Groningen | The Netherlands |
| Carmen | Estrada | Clinica Tejerina | Madrid | Spain |
| Eva M. | Fallenberg | Charité Universitätsmedizin Berlin | Berlin | Germany |
| Gábor | Forrai | Duna Medical Center | Budapest | Hungary |
| Magaly | Garza | San Jose Tec | Monterrey | Mexico |
| Fiona J. | Gilbert | Addenbrookes | Cambridge | United Kingdom |
| Pål Erik | Goa | NTNU | Trondheim | Norway |
| Ahuva | Grubstein | Beillinson Hospital | Tel-Aviv | Israel |
| Brian | Hargreaves | Standford University | San Francisco | United States of America |
| Thomas H. | Helbich | Medical University of Vienna | Vienna | Austria |
| Nola | Hylton | UCSF | San Francisco | United States of America |
| Mami | Iima | Kyoto Universtiy | Kyoto | Japan |
| Blanca | Kanagusico | San Jose Tec | Monterrey | Mexico |
| Sungheon Gene | Kim | NYU | New York City | United States of America |
| Karen | Kinkel | Les Grangettes | Switzerland | Switzerland |
| Christiane K. | Kuhl | University Klinik Aachen | Aachen | Germany |
| Sibel | Kul | Karadeniz Technical University | Trabzon | Turkey |
| Denis | Le Bihan | NeuroSpin, Frédéric Joliot Institute | Gif Sur Yvette | France |
| Marc | Lobbes | Maastricht UMC+ | Maastricht | The Netherlands |
| Agnes | Malgouyres | Siemens | Paris | France |
| Ritse M. | Mann | Radboud University Nijmegen Medical Center | Nijmegen | The Netherlands |
| Laura | Martincich | Candiolo Cancer Institute, FPO - IRCCS | Candiolo (TO) | Italy |
| Elizabeth | Morris | MSKCC | New York City | United States of America |
| Utaroh | Motosugi | University of Yamanashi | Yamanashi | Japan |
| Linda | Moy | NYU | New York City | United States of America |
| Marcelo | Muñoz | CIM | Rosario | Argentina |
| David | Newitt | UCSF | San Francisco | United States of America |
| Savannah | Partridge | University of Washington | Seattle | United States of America |
| Andrew | Patterson | Addenbrookes | Cambridge | United Kingdom |
| Federica | Pediconi | Sapienza University of Rome | Rome | Italy |
| Hans | Peeters | Philips | Best | The Netherlands |
| Elsa | Perez | Hospital Josep Trueta | Girona | Spain |
| Silvia | Pérez | MD Anderson Cancer Center | Madrid | Spain |
| Katja | Pinker-Domenig | MSKCC, Medical University of Vienna | New York City | United States of America |
| Miguel Angel | Pinochet | Clinica Alemana | Santiago de Chile | Chile |
| Habib | Rahbar | University of Washington School of Medicine | Seattle | United States of America |
| Yael | Rapson | Beillinson Hospital | Tel-Aviv | Israel |
| Javier | Rodriguez | Clinica Rosario | Rosario | Argentina |
| Allison | Rose |  | Melbourne | Australia |
| Francesco | Sardanelli | University of Milan, IRCCS Policlinico San Donato | Milan | Italy |
| Anabel M. | Scaranelo | University of Toronto / Princess Margaret Cancer Centre-UHN & MKBC Sinai Health System | Toronto | Canada |
| Simone | Schrading | University Klinik Aachen | Aachen | Germany |
| Tamar | Sella | Hadassa Hospital | Jerusalem | Israel |
| Nisha | Sharma | Leeds Teaching Hospital | Leeds | UK |
| Eric E. | Sigmund | NYU Langone Health | New York | United States of America |
| Miri | Sklair-Levy | Sheba Medical Center | Ramat Gan | Israel |
| Julie | Soens | Kliniek St Jan | Brussels | Belgium |
| Rafaela | Soler | Complejo Hospitalario Universitario A Coruña | A Coruña | Spain |
| Alejandro | Tejerina | Clinica Tejerina | Madrid | Spain |
| Sunitha | Thakur | MSKCC | New York City | United States of America |
| Fabienne | Thibault | Institut Curie | Paris | France |
| Isabelle | Thomassin-Naggara | Hospital Tenon | Paris | France |
| Bruno | Triaire | Olea Medical | La Ciotat | France |
| Rubina M. | Trimboli | University of Milan | Milan | Italy |
| Mireille | Van Goethem | University Hospital Antwerp | Antwerp | Belgium |
| Astrid | Van Hoyweghen | University Hospital Antwerp | Antwerp | Belgium |
| Chantal | Van Ongeval | University Hospitals Leuven | Leuven | Belgium |
| Elisabeth | Weiland | Siemens Healthineers | Erlangen | Germany |
| Thomas | Yankeelov | University of Texas Austin | Austin | United States of America |

**Appendix 2: List of items reviewed for the consensus that achieved 80% agreement**

|  | **A. Recommended parameters (minimum quality standard)** |
| --- | --- |
|  | Axial orientation |
|  | Acquired (physical, not reconstructed) in-plane resolution 2x2mm²  or smaller |
|  | FOV to cover both breasts |
|  | Fat suppression |
|  | DWI sequence with at least 2 b values |
|  | Lowest b value as close to 0 as possible, but not larger than 50s/mm² |
|  | High b value = 800 s/mm² |
|  | Calculate ADC maps using DWI images at low b and high b, as: ADC=ln(Slow/Shigh) / (bhigh-blow) where Slow,high are the image signal values obtained with b values blow,high |
|  |  |
|  | **B. Hardware** |
|  | Whole-body >=1.5T scanner |
|  | Gradient hardware capable to reach >=30mT/m |
|  | Dedicated breast receive-only coil with ≥4 channels |
|  |  |
|  | **C. Quality control (QC)** |
|  | Fat suppression efficiency (need to define how to evaluate) |
|  | Amount of ghosting (need to define how to evaluate) |
|  | Signal/noise level (need to define how to evaluate) at lowest b value |
|  | Signal/noise level (need to define how to evaluate) at high b value |
|  | Level of stability/reproducibility (need to define how to evaluate) |
|  | Perform quality control on human volunteers |
|  | Quality control of image processing pipeline (including preprocessing and ADC calculation) |
|  | Routine quality control mandatory |
|  | Quality control after each maintenance |
|  | Quality control after each upgrade or change in hardware/software |
|  |  |
|  | **D. Acquisition parameters** *(note: those parameters values might depend on MRI hardware and software, but must be chosen so that requirements set in section A are met)* |
|  | SPAIR as the preferred fat suppression method |
|  | Reconstructed resolution 2x2mm² or smaller |
|  | EPI readout as the Diffusion MRI encoding scheme |
|  | Minimum TE allowed by the MRI system and choice of parameters |
|  | Receiver Bandwidth (rBW) optimized to produce a minimum TE. |
|  | Acceleration: GRAPPA or SENSE (note: will reduce SNR) |
|  |  |
|  | **F.      Analysis, interpretation and reporting** |
|  | Morphological assessment requires both high b DWI and CE MRI images |
|  | Lesion classification requires use of high DWI/ADC images and CE MRI |
|  | Mean quantitative ADC value (10-3mm/s²) must be reported |
|  | ROIs must contain at least 3 voxels |
|  | Necrotic or hemorrhagic areas must not be included in ROI/VOI |
|  | Unrealistic (very low/high or negative ADC values) requires the ROI/VOI to be repositioned |
